# One or many labels? a longitudinal qualitative study of patients’ journey to diagnosis at a specialist NHS Postural Tachycardia Syndrome (PoTS) clinic

**DOI:** 10.1371/journal.pone.0302723

**Published:** 2024-07-10

**Authors:** Iris Knoop, Annie S. K. Jones, Ereza Ibrahimi, Angeliki Bogosian, Nicholas Gall, Rona Moss-Morris

**Affiliations:** 1 Institute of Psychiatry, Health Psychology Section, Psychology and Neuroscience, King’s College London, London, United Kingdom; 2 School of Health and Psychological Sciences, City, University of London, Northampton Square, London, United Kingdom; 3 Cardiology Department, King’s College Hospital, London, United Kingdom; University of Ghana College of Humanities, GHANA

## Abstract

**Objectives:**

Postural Tachycardia Syndrome (PoTS) is a poorly understood syndrome of multiple disabling symptoms. This study explored the process of seeking a diagnosis of PoTS. Analysis focused on changes before and after participants’ first appointment with a national PoTS clinic, and explored whether a diagnosis is beneficial in the context of multiple co-occurring conditions and an absence of licenced treatments.

**Design:**

A longitudinal, qualitative study.

**Methods:**

Participants (*n* = 15) in this nested qualitative study were recruited from a larger study of people who had been newly referred to a National specialist NHS Cardiology PoTS service. Semi-structured interviews were conducted remotely before, and 6 months after their first appointment with the clinic. Data was analysed longitudinally and inductively using Reflexive Thematic Analysis.

**Results:**

Three overarching themes were identified: *“Slowly moving forward and finding positive gains”*, *“Needing more pieces of the puzzle to see the bigger picture”*, and *“The value and impact of investigations”*. Findings suggested that not much had changed in the 6 months between interviews. Participants were moving forward in terms of diagnoses, treatment and adjustment following their appointment, but many were still seeking further clarity and possible diagnoses. Investigations, appointments, and new-found problems, continued to have a substantial impact over time.

**Conclusions:**

The journey to diagnosis for patients with suspected PoTS appeared to promote acceptance of self, and of limitations posed by symptoms. However, many participants continued their search for an explanation for every symptom experience, and this may become increasingly complex, the more labels that have been acquired. Lack of clarity contributed to ongoing difficulties for this patient group alongside fraught relations with health care professionals (HCPs). A more coherent, integrated approach which is communicated clearly to patients is recommended.

## Introduction

Postural (Orthostatic) Tachycardia Syndrome (PoTS) is a relatively recently defined [1] syndrome of multiple disabling symptoms [2,3] which tends to occur more commonly in women (>80%) who are of childbearing age. Although the exact prevalence of PoTS is unknown, pre-Covid-19-pandemic estimations were 0.2%-1.0% in developed countries [4]. There are currently no licenced treatments for PoTS, but there is a small but emerging evidence base for pharmacological and non-pharmacological treatments which may provide benefit [4–6]. The aetiology of PoTS remains unclear; recent research proposes several potential mechanisms such as autoimmune bases, hypovolaemia with reflex tachycardia, small fibre neuropathy, and hyperadrenergic states [7]. Symptoms are often precipitated or exacerbated by an event such as a viral infection, physical or psychosocial trauma [8], pregnancy, vaccination, or surgery [7], although gradual onset can also occur. PoTS is diagnosed based on a persistent orthostatic heart rate increase of >30bpm in the first 10 minutes of being in the upright position, at least 3 months of chronic orthostatic intolerance, the absence of orthostatic hypotension, and absence of other diagnoses that could cause similar symptoms [9,10]. Symptoms of PoTS can vary but tend to include orthostatic symptoms (such as palpitations and dizziness), wider autonomic symptoms (for example relating to the gut and bladder), and non-orthostatic symptoms (such as cognitive dysfunction, fatigue, insomnia, or headaches). The broad variety and complexity of symptoms and commonly co-occurring conditions which may provide overlap in symptomatology [10], combined with a lack of healthcare provider education & awareness, as well as enduring disbelief, have contributed widely to diagnostic delays and misdiagnoses, with many patients struggling for years to receive a diagnosis [11,12].

PoTS is for many people a long-term cause of disability and impaired quality of life, and is associated with depressive symptoms [6]. The path to finding the right specialist can be fraught with complexity, requiring all clinicians in the chain of referral to recognise and support the need for referring patients presenting with this wide-ranging constellation of symptoms and co-occurring conditions. Furthermore, in the UK and likely elsewhere, there is a paucity of specialist healthcare service provisions for the condition, and receiving the correct care and a diagnosis requires significant persistence on the part of the patient [13]. Although the understanding and evidence base regarding PoTS pathophysiology and therapeutic approaches has expanded in recent years, like in many other medically uncertain conditions [14–17], an ambivalence remains amongst some healthcare professionals (HCPs) regarding the legitimacy of PoTS [18]. HCPs not recognising PoTS, and the ensuing lack or delay of diagnosis can have negative consequences on patients’ self-management, adjustment, and ultimately wellbeing [13,18–25].

A diagnostic label is considered important for guiding management and coping mechanisms [26], to inform treatment plans and further investigations, or to indicate the absence of other, more serious disease [27]. However, in the context of poorly defined and understood conditions, there is some evidence to suggest that the benefit of certain diagnostic labels (for example of syndromes such as Fibromyalgia) may be limited [28–32]. Reasons for this are not entirely certain, but might relate to a lack of clarity around aetiology or treatment implications, or the psychiatric, social, and medical meaning or stigma attached to certain labels. Where there are multiple co-occurring conditions, it is also not known at which point additional labels cease to make meaningful contributions to therapeutic approaches, legitimacy, or coherence for patients. Longitudinally studying the dynamic process of obtaining a diagnosis of an incompletely understood condition such as PoTS could provide an important starting point for understanding how helpful such a diagnosis is perceived to be by the patient.

Previous qualitative studies have explored the lived experience of people with PoTS [18,33]; however the current study is the first to qualitatively investigate the diagnostic process over time by interviewing patients with suspected PoTS before diagnosis and 6 months after their first diagnostic consultation. This study aimed to explore patients’ experiences and reported self-management of their symptoms before, and 6 months after their first appointment with a specialist PoTS clinic, with a view to understanding how these change over time and what impact, if any, the diagnosis of PoTS may have.

## Methods

Ethical approval for the study was granted on 27/10/2021 by HRA and Health and Care Research Wales (HCRW) and the London-Surrey Research Ethics Committee (Research Ethics Committee reference: 21/LO/0728). The current study is reported following the Standards for Reporting Qualitative Research (SRQR) [34].

### Patients and public involvement

This study was part of a larger mixed-methods project investigating correlates and predictors of symptom severity over time in people with PoTS. Fourteen patient & carers from the PoTS patient charity PoTS UK gave feedback on the initial conceptualisation of the study, the questionnaires used, and the burden of participation in the wider study. Feedback was sought on preliminary themes from the trustee and chair of PoTS charity PoTS UK.

### Materials

Key demographic and clinical data were collected from participants’ questionnaires. Participants’ medical records were accessed with participant consent in March 2023 after follow-up interviews were completed to extract current diagnostic status in regard to PoTS. Participants’ average self-reported orthostatic symptom burden was measured with the Orthostatic Grading Scale (OGS) [35] which can be used to classify symptoms of orthostatic intolerance as mild <4, moderate 4–9, or severe >9 [36]. Average self-reported distress scores were assessed with the Kessler psychological distress score (K10) [37] which classifies scores <20 as well, 20–24 as mild, 25–29 moderate, and >30 severe mental disorder [38].

### Participants

Participants in this nested qualitative study were recruited between 11/2021 and 12/2022 as part of a larger, single-centre, mixed-methods longitudinal project conducted in a UK specialist National Health Service (NHS) PoTS clinic. 465 consecutive patients on the national clinic waiting list were sent a letter within one month of their first appointment to invite them to take part in the wider questionnaire-based study, which participants accessed and gave written consent to online. This then provided participants with an option to take part in the qualitative interviews, from which eligible participants were selected through a mixture of convenience and purposive sampling until the current target sample size and data saturation was reached.

Eligibility criteria for participation in this study included: (1) having a suspected but unconfirmed diagnosis of PoTS; (2) being older than 18 years of age; and (3) being able to speak English. Participants were interviewed prior to their specialist appointment (Time 1 (T1) between 11/21-05/22) and at 6 months follow-up (Time 2 (T2) between 04/22-11/22).

Patients are referred to this neuro-cardiology clinic for specialist assessment in order to establish or confirm a PoTS diagnosis according to current diagnostic guidelines [7] (an orthostatic HR increase of >30bpm with orthostatic symptoms) and receive specialist treatment for PoTS/orthostatic intolerance. The clinic is run by a cardiologist specialising in PoTS and supported by a nurse-led arrhythmia service, and referrals to the clinic are made by clinicians based on suspicion of PoTS with PoTS-like symptoms and/or suggestive tilt-table test results. Patients cannot self-refer to this NHS clinic. The waiting time between referral and first appointment with the clinical lead is approximately 12 months, during which time testing (tilt-table test or active stand, cardiopulmonary exercise testing, Holter monitoring, echocardiogram, and blood tests [35]) commences. After the initial clinic appointment, further test results and treatment responses are discussed in annual telephone follow-up appointments. Where required, patients are referred on to other specialisms or for additional testing and treatment.

### Interviews

Semi-structured interviews were conducted via video link by the first author (IK). Interviews were audio recorded, transcribed verbatim, and de-identified. The topic guide for T1 and T2 can be found in S1 Appendix.

### Data analysis

The analysis was conducted through Relativist epistemology and Critical Realism ontology lenses, which recognise that truth and reality are inevitably shaped by human experience [39]. Data was analysed inductively by working iteratively with the phases mapped out by Braun and Clarke for Reflexive Thematic Analysis [39]. Transcripts were read repeatedly and line-by-line, unstructured coding was completed in NVivo (version 12.7) by two coders (EI and IK). Our inductive approach enabled the development of themes to be driven by data rather than deductively linking back to concepts, pre-existing coding frames, or researchers’ analytic pre-conceptions.

**Fig 1:**
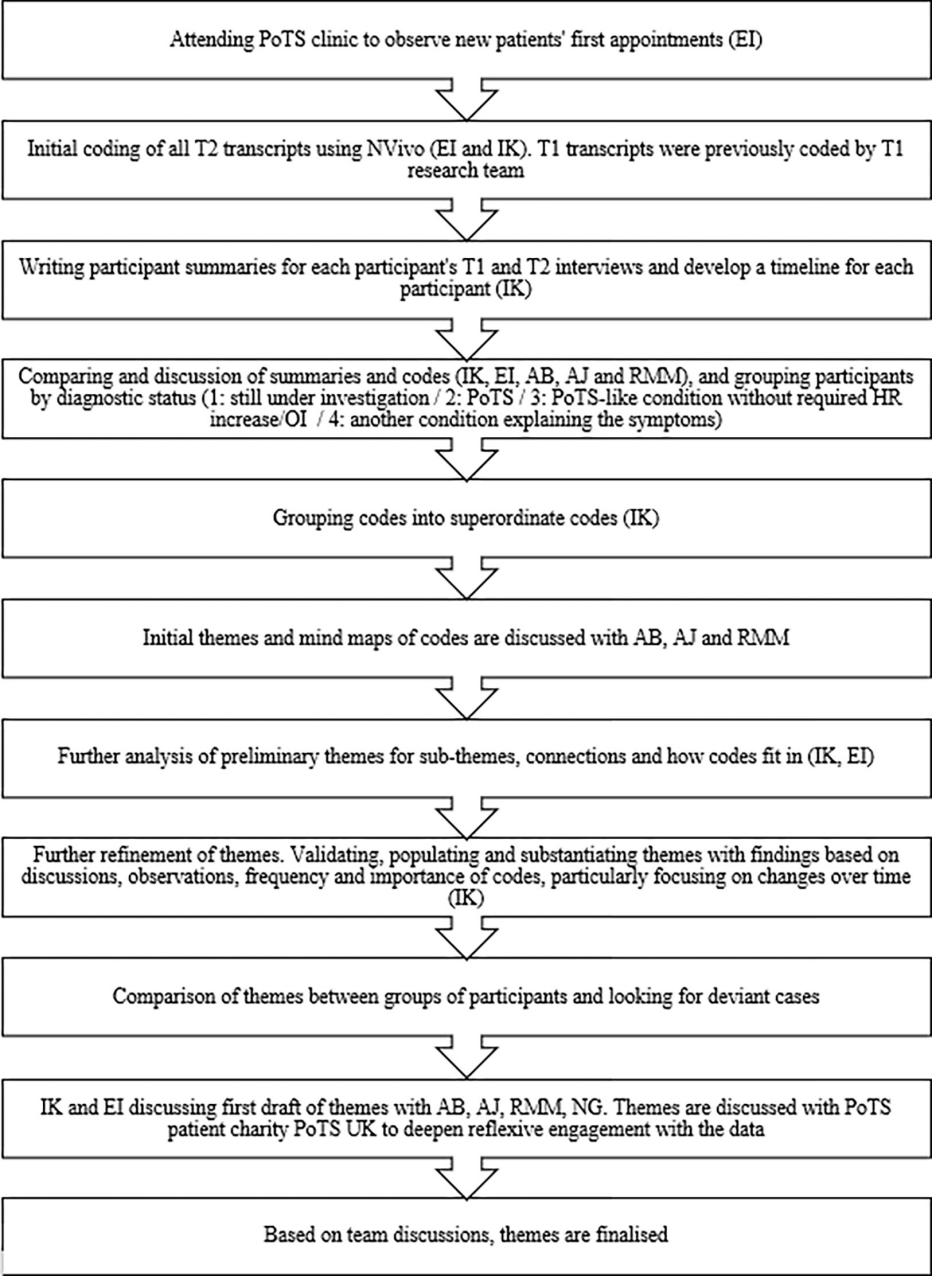
Data analysis flow chart. *Note*: PoTS = Postural Orthostatic Tachycardia Syndrome; OI = Orthostatic Intolerance; HR = Heart Rate.

Overarching codes and central themes were compared between baseline (T1) and 6-month follow-up (T2). Participant summaries were written for each participant, detailing a timeline, changes from T1 to T2 interviews, and T1&2 symptom and distress scores. The reflexive analytic process reported is outlined in Fig 1.

## Results

### Participants

A summary of participant demographics can be found in Table 1. Participants’ average self-reported symptom burden and distress was similar at baseline and follow-up, indicating severe orthostatic symptom burden and mild distress. Medical records showed that by follow-up, only four participants had been definitively diagnosed with PoTS, seven did not quite meet heart rate criteria and were diagnosed with Orthostatic Intolerance (OI, “PoTS-ie” without sufficient orthostatic heart rate increase), three were still under investigation, and one received an alternative diagnosis. Resource constraints at this clinic mean that heart rate is assessed during appointment availability which may be at any time of day. Diurnal variation [40–42] of the orthostatic heart rate response is therefore not taken into consideration and this is a limitation of current PoTS treatment in the UK. However, treatment approaches at this clinic would be similar for patients whether they received a diagnosis of OI or PoTS.

**Table 1:** Participant characteristics *n* = 15 (T2).

	Number (%) / Average
Age	39 ± 12.7, range 22–58
Gender	
• Female	12 (80%)
• Male	2 (13%)
• Non-binary	1 (7%)
Ethnicity	
• White	13 (87%)
• Chinese	1 (7%)
• Mixed white/Asian	1 (7%)
Duration of symptoms	13.4 ± 15.3 years, range 1.5–40
Patient-reported PoTS diagnoses at T2 interview	4 (26%)
Diagnosis from PoTS clinic as per medical records at time of analysis:	
• PoTS HR criteria met	4 (26%)
• PoTS-like symptoms without sufficient Heart Rate increase (‘PoTSie’ / PoTS-like / Orthostatic Intolerance)	7 (46%)
• Still under investigation	3 (20%)
• Other diagnosis	1 (7%)
Symptom severity (OGS; range 0–20) T1	10.3 ± 3.2
• T2	11.4 ± 3.5
Distress (K10; range 10–50) T1	21.8 ± 7.7
• T2	21.4 ± 6.1
Occupational status:	
• Full time employed	6 (40%)
• Unemployed—due to PoTS	4 (27%)
• Unemployed—unrelated to PoTS	2 (13%)
• Home maker	1 (7%)
• Student/training—part time by choice/other reason	1 (7%)
• Other	1 (7%)

*Note*. PoTS = Postural Orthostatic Tachycardia Syndrome. OGS = Orthostatic Grading Scale. K10 = Kessler Psychological Distress Scale. T1 = Time one (first) interviews. T2 = Time two (follow-up) interviews.

### Overarching themes

Analysis resulted in three superordinate themes: “*Slowly moving forward and finding positive gains*”, “*Needing more pieces of the puzzle to see the bigger picture*”, and “*The value and impact of investigations*”. Illustrative quotes are from T2 interviews, unless specifically stated that they are from T1 interviews. A summary of the themes and central concepts over time can be found in Table 2 and are detailed below. A more detailed coding tree for T2 interviews can be found in S2 Appendix.

**Table 2:** Summary of themes and central concepts from T1 to T2 interviews.

Baseline interviews (T1)		Follow-up interviews (T2)		Notable changes over time
Theme	Central organising concepts	Theme	Central organising concepts	
**Seeking physiological coherence and validation**	Understanding symptoms and physiology Seeking validation from HCPs Awareness of PoTS among HCPs	**Slowly moving forward and finding positive gains**	Benefits from appointment or diagnosis Improvements Retrospection	This theme centred around positive gains over time following their appointment; legitimacy, reappraisal, and acceptance, leading to coping by focusing on symptoms and placing limitations. Participants had a positive experience in clinic which appeared to rebuild confidence in the healthcare system. Some participants got the hoped for validation, which had positive benefits of how people saw themselves. Acceptance led not only to acceptance of self, but also of their limitations.
**Individual persistence**	Pushing for referrals and investigations Pushing themselves Acceptance vs striving	**Needing more pieces of the puzzle to see the bigger picture**	Connecting more dots Lack of clarity Negative HC experiences Questioning HC and self-management strategies	Despite receiving some ‘answers’ at T2, these took time and did not always provide satisfactory coherence. The pursuit of missing pieces of the ‘personal health puzzle’ persisted over time for many participants. Lack of clarity and communication difficulties may have compounded previous negative HC experiences, with participants over time continuing to display low levels of trust, or questioning information they were given.
**Navigating the cumulative burden**	The accumulative effect of symptoms from multiple conditions, psychosocial impact, and burden of investigations & treatment Coping with the impact of the condition Uncertainty Diagnostic difficulties	**The value and impact of investigations** **(and the ‘*cumulative burden*’, continued from T1 analysis)**	The value of proper testing Impact of investigations Finding new problems	The cumulative burden of symptoms, co-occurring conditions, investigations, treatment, and psychosocial impact remained high over time. Thorough investigations provided reassurance and legitimisation. However, ongoing appointments and finding additional health problems negatively impacted some participants. Previous adverse experiences, uncertainty, and/or diminished trust levels appeared to extend into participants’ questioning of certain tests and/or results.

*Note*. HC = Healthcare, HCP = Healthcare professional.

#### Slowly moving forward and finding positive gains

Generally participants, including those who perhaps did not receive the diagnosis they were hoping for, described their appointment in the specialist PoTS clinic as a positive experience. They described feeling listened to and taken seriously by a receptive specialist who understood the condition and associated health issues, and proactively addressed these:

the appointment with [Doctor] was different than really any appointment I had before then. In that, usually I’d be the one trying to figure out my own diagnoses or like doing the research or whatever. But he was offering me all this information and pointing out things to me about myself that I haven’t noticed…normally was completely the other way around. (Participant A)

A sense of validation and relief were some of the main positive outcomes for participants who received a diagnosis.

…it’s just that kind of very intense relief, gratefulness, understanding, vindication, I wasn’t making it up, it’s a real thing. I cried, on the way out, but just relief. Just I’m validated. (Participant B, T2 interview)

This was a notable change from T1 experiences of self-doubt:

I feel such a fraud at the moment, going to all these appointments… I just feel like a bit of drama queen really… (Participant B, T1 interview)

There was acknowledgement that a diagnosis may not necessarily change the impact of the condition, but the explanations helped to make sense of things, increased self-acceptance and decreased self-blame.

I think it means, apart from the fact that I can stop feeling guilty about not being able to do stuff, or feeling sort of lazy, or stupid or, uncapable or anything like that. I know nothing is changing, and I’m still going to be tired and all of those sorts of things. But at least knowing there’s a reason, and I don’t just need to try harder, that there is actually something physically wrong, means I can stop blaming myself…So, the other, it’s just everything is making sense. (Participant B)

Participants described feeling hopeful after their appointment, irrespective of the outcome of investigations. For some this was despite expressing at T1 that they had lost trust in the medical profession.

I feel hopeful that [Doctor] will actually find a solution. And it’s possibly the first time that I’ve actually been hopeful. I’m sort of positive that they’re looking into it, but not positive that they’ll find something…I just, I would just like some sort of solution so I can carry on with my life, because this is not fun. (Participant C)

In addition, at T2, despite many not yet having received a formal diagnosis, there was some sense of coping and managing better. One common coping strategy was being aware of, recognising, and staying within their limits, to prevent deterioration:

And when I’m feeling good, then I know how to manage this now… I understand what my limitations are now, and I try and keep within them so I don’t spark another downturn, shall we say. (Participant C)

Others described how they changed previous ways of coping such as pushing too hard or boom-and-bust behaviour:

I think before I was really pushing myself and really trying to exercise my way out of ill health and it was completely the wrong thing. It was just like crash, crash, crash, just boom and bust…it’s just been so confusing up until I started to actually get, move towards diagnosis (Participant D)

Others at T2 had continued the same self-management strategies as before diagnosis, but described having a clearer understanding of their broader symptoms and conditions, and learning new management techniques for these:

I think having the diagnosis didn’t really change them [self-management strategies] like I already knew that they helped…I think for me PoTS is just one part of the picture of my chronic illness and that’s been kind of confirmed over the past year…so it’s been really good to kind of get those pinpointed and learn different management techniques for different things…So that’s been really helpful as well.” (Participant A)

Improvements were attributed to various strategies and factors, including pacing & pre-planning, antihistamines, breathing exercises, diet, exercise, medication, resting, salt, water, mindfulness, yoga, meditation, self-kindness, time, but also, listening to and understanding their body and heeding any warning signs:

More of an understanding of the illness, probably being medicated. And then there’s things I do, sort of self-help type things.… I still have to be careful and pace myself…sort of listening to my body as well. I’m a lot better at saying no to things than I was before… just learning to, sort of, live your life in a certain way… I know that I’m tired if my hands start to shake, or I’ve got tinnitus… So, my body gives me signals as well, which doesn’t sound very nice but is actually quite helpful. (Participant F)

Although somatic-focusing was observed across T1 and T2 interviews, there were varying approaches. Some described bodily vigilance as initially feeling counterintuitive but that it helped them to start recognising the signs and being aware of their limitations. Pre-empting a downturn appeared to be an important part of self-management, despite feeling conscious of being perceived by others as hypervigilant towards physical sensations.

I’m always worried about being a hypochondriac, because it feels like that’s what people are labelling you. But I think in pacing you have to become really self-aware, and you have to scrutinise your body quite intensely, which, so it feels a bit counterintuitive to that like instinct to try and not be a hypochondriac. But you have to really notice in your body like what is going on so you can start to see the signs of things starting to go downhill before they actually do. (Participant A, T1 interview).

Others described a contrasting approach at T1, where they tried to divert their focus away from health issues to minimise the impact on their lives:

I’ve gone to a point where I do want to like life as best as I can, embrace life…Health is still going on. It’s still doing what it’s doing, but I’m not—It’s not just my main thing. I’ve got other things going on which are quite nice. And I’m thankful for that. It was needed…Because I—I lived health, chasing specialists, trying to get answers, trying to get appointments sooner rather than later…(Participant G, T1 interview)

Despite their initial attempts to not let health ‘take over’ their life, in follow-up interview, the impact of not fully meeting diagnostic PoTS heart rate criteria was significant for this participant and left them feeling uncertain:

I stupidly put so much emphasis and hope into, because I really thought that PoTS was going on. You know, it felt like it was the last piece of the jigsaw, especially having hypermobile EDS. You know, I understand it kind of goes together. So now I feel like I’m in limbo in terms of okay, I don’t meet the criteria, however, I am PoTSie. So where does that leave me exactly? (Participant G)

This also illustrated that even when some ‘answers’ were provided, at times this raised more questions for participants, re-iterating the need for coherence and clarity which will be discussed in more detail in the next theme.

#### Needing more pieces of the puzzle to see the bigger picture

Despite participants finding some ‘pieces of the puzzle’ on their journey to diagnosis, many were still seeking coherence at T2. This search was not contingent upon receiving a diagnosis; for many, this led to a continued search for more labels, or further clarity.

I really want to look into…ADHD…I know it’s increased in the hyperadrenergic PoTS as well…I’m pretty excited. Unfortunately, I’m then sort of focusing a lot on it… I don’t know who to ask to get an ADHD assessment organised…so it carries a bit more weight and urgency, because I hear there’s like, years waiting list… I could just leave it and see, but I quite like labels, I think, helps understand. (Participant B)

Participants placed different value on receiving a PoTS label. While some participants expressed at T1&2 that a diagnosis would give them tangible legitimacy, others appeared less concerned about whether they would receive a diagnosis, and some were prepared that a diagnosis might not be helpful. Having a collection of diagnoses was not always considered useful. For example, some participants with Hypermobile Ehlers Danlos syndrome (EDS), or chronic fatigue syndrome / myalgic encephalomyelitis (CFS/ME) questioned the helpfulness of these diagnoses.

I don’t really know whether it will help, to have an official diagnosis [of PoTS]…obviously it’s important to know what’s going on. But what I found with the EDS is that it’s sort of, it doesn’t solve it. (Participant D)

I was diagnosed with ME like several years before, and that wasn’t a particularly reassuring diagnosis at the time because, like the NHS really don’t have much to offer for that…it was quite a depressing diagnosis (Participant A)

Some participants, however, experienced disappointment at not receiving a diagnosis of PoTS by T2, despite being offered treatment and onward referrals for their symptoms. This illustrated that for some, coherence and a diagnostic label were more important than treatment.

I didn’t get a diagnosis of PoTS which was, I’m gutted about to be honest with you. Because, not in the sense of wanting more on my health records, but to kind of make sense and name something in terms of what the hell is going on. (Participant G)

Interestingly, it was not always those who were most affected or disabled by their symptoms who met PoTS diagnostic HR criteria. One participant had been aware of their high heart rate for years but was not experiencing particularly disabling symptoms. They met PoTS HR criteria and received a medical diagnosis of inappropriate sinus tachycardia (IST) with BP variability and orthostatic intolerance, highlighting further complexities between the HR, PoTS, and other related and overlapping conditions. In contrast with many patients with suspected PoTS, they had not seen anyone else for their symptoms.

Because I think I don’t have problems, it doesn’t sort of interfere. I don’t get dizziness. I don’t get out of breath. It doesn’t make me feel uncomfortable. So I just kind of tolerate it and carry on with my day…The only reason why I went down sort of trying to get the diagnosis route so that I knew that it wasn’t something that I should be concerned about long term. (Participant H)

Others who were strongly affected by symptoms suggestive of PoTS, did not always meet the diagnostic heart rate criteria. The uncertainty left them without an understanding of the cause of their further declining health and decreasing independence, which they found difficult to explain, particularly at work.

I think a diagnosis for PoTS would be great, because it’s just something that I can tell people, rather than them saying, Oh, what’s up? And I just have to list my symptoms…For work, it would be really helpful because obviously I’m taking a lot of sick days, but I don’t have any solid proof that I’m actually ill. (Participant I)

A diagnosis was seen as providing a common understanding and explanation for their symptoms. Providing this coherence and clarity to others appeared to be an important goal of the sense-making process, in order to receive the understanding and support participants required. Some also felt they needed a diagnosis before starting self-management.

I’d prefer a formal diagnosis. It just makes it easier, doesn’t it. You can’t go to someone and say, Oh, I suffer with PoTS symptoms, but you can go and say I suffer with PoTS. It is very different, so I don’t know and I haven’t started increasing salt or anything like that because I don’t know what effect that would have in my blood pressure…And it was like it’s just so confusing. It really is. (Participant J)

For some, there was hesitancy around trying new medications, perhaps connected to previous adverse experiences, or generally feeling wary of taking medication. There was also some scepticism regarding non-pharmacological self-management strategies such as drinking more water. This appeared to be due to lack of clarity and consensus on what constituted ‘proper medical advice’ received from HCPs, with several participants highlighting that more consistent, robust, evidence-based guidance would be helpful. A sense of mistrust was also palpable.

But there is no consensus if you like, right? So that’s what is kind of worrying. It feels like more an opinion than, like an actual diagnosis. And I think that’s what I was, I’m kind of struggling with, trust, basically. I don’t really trust [laughs] any of them. (Participant K)

#### The value and impact of investigations

The cumulative burden of symptoms, appointments, investigations, treatments observed at T1 remained high at T2. Because the baseline study detailed this cumulative burden extensively, the current analysis focused on participants’ views of the value and impact of medical appointments, diagnoses, and investigations, and any changes observed within this cumulative burden.

Thorough testing provided legitimisation and reassurance by ruling out more dangerous health and cardiac problems. It helped participants to feel taken seriously, and to feel validated when test results corroborated their symptoms.

I was relieved that I was having all these tests done and that it’s showing up on the tests as well. Whereas before I go to the doctors and they’d be like, Oh, yeah, It’s [heart rate] high, But it’s probably just anxiety, or push it off as another thing. Whereas in the actual tests that they’re doing, the different ones, you can see how bad it actually gets. (Participant L)

At times this came after many attempts of seeking help.

I went to several doctors whilst I was training, because the fatigue was the worst part of it… I was told that it was because I’d put on weight, and that it was just more difficult for me to lift my limbs, because I put on weight. Bearing in mind, I’ve been previously anorexic, and that’s why I put on weight, so that was fun… they didn’t focus on any of my PoTS symptoms at all, despite the fact that that’s what I was referred there for. (Participant I, T1 interview)

Participants generally valued having their broad range of symptoms investigated ‘properly’, and being referred where necessary, even when they may not have met formal criteria or presented as ‘classic PoTS’:

he referred me to endocrinology, and he referred me to respiratory physio and stuff like that. And it was just all such like, I don’t know. It was just so nice to be helped like in ways that I haven’t even needed to push for or even anticipated, that something like that might be helpful. (Participant A)

It was clear at T2 that while finding out about additional health problems (e.g. bladder issues, reactive hypoglycaemia) from thorough investigations was generally viewed as helpful by participants, this was sometimes unexpected. Some experienced the process of finding multiple additional health problems in medical testing as ‘relentless’:

But also now they found that I have really high prolactin in my blood system, and now they think I’ve got a little prolactinoma in my brain, and then they discovered I had [laughing]—every time they do blood tests, basically, they’re finding more problems, so it just feels relentless… it is a bit like drowning basically. (Participant M)

The need to adjust to new health issues therefore had the potential to add further to their cumulative burden.

I have to self-catheterise now, in every morning and every night…I was more shocked about the urology, so I was kind of surprised. I mean, I know it’s affected me,…But… I wasn’t aware like that there was anything they could do to treat it. I thought it would be more like, You’ve got it, There’s not a lot we can do. So it’s just, at the moment trying to get the hang of it so that I’m more comfortable with using it, and then it should help me long term…it has been quite stressful. (Participant L)

Existing PoTS management strategies also had the potential to clash with the management of these newly identified bladder problems:

I do feel a lot better when I’m hydrated. But the hard thing is that it makes me feel better as in myself, and my heart rate when I’m hydrated, and the dizziness as well helps with the hydration. But it’s hard on my bladder because drinking all of that and then having the bladder symptoms at the same time means if I feel better myself, but I’m going to the toilet every 10 minutes. (Participant L)

As well as the results, the tests themselves could be taxing. One participant still under investigation at T2 explained the difficulties in completing some of the tests, particularly those which had the potential to bring on or exacerbate symptoms.

I don’t travel very well at all. I don’t like hospitals anyway. But you feel exhausted for days afterwards…I react to most things…I’m just not happy about being injected with something that could potentially make my heart go off the scale…(Participant N)

For those still in work, investigations and clinic appointments for various conditions required frequent time off work or using annual leave to attend appointments:

I prefer to go where the expertise is don’t get me wrong, but I think God it’s more like a whole day off of work…It’s not just the day…But it’s the next day you’re just going to feel absolutely wiped out. (Participant E)

The diagnostic tilt table test (TTT) or active stand test, which are used interchangeably to diagnose PoTS, appeared to be a particular source of concern. One participant recounted being asked due to a communication error to perform an active stand instead of a TTT, despite being a wheelchair user, while several others questioned whether this test was being conducted in optimal circumstances to demonstrate the orthostatic heart rate increase required for a diagnosis. There was a tangible sense of urgency in several participants’ interviews, suggesting that delays and waiting times for appointments and results were added difficulties.

## Discussion

Participants’ timelines revealed that investigations for PoTS can be prolonged, and for many, little had changed during the 6 months from the baseline interviews. The significant delays from diagnosis to follow up and further management likely influenced the feelings of frustration and uncertainty. Participants spoke about some positives. Being seen in the PoTS clinic by a specialist who legitimised their condition helped foster acceptance of the limitations posed by the symptoms, and a more positive self-image. It also provided some welcome relief from co-ordinating their own care and unhelpful healthcare interactions, and helped participants come to terms with negative past experiences where others may have dismissed their illness.

However, many were still seeking further clarity and understanding in relation to their health. Investigations, appointments, and new-found problems, also continued to have a substantial impact on some participants and the overall cumulative burden of symptoms, multiple long-term conditions, and their psychosocial impact.

At 6 months follow-up interviews only 4 participants (26% of those interviewed) had received a definitive PoTS diagnosis, 7 (46%) were diagnosed as PoTS-like without sufficient heart rate increase, 3 (20%) were still under investigation without a diagnosis, and 1 (7%) received an alternative explanation for their symptoms. Despite contrasts between individual participants, there were no clear differences between diagnostic groups. Those with a firm PoTS diagnosis did not always appear nearer the end of their investigative health journey than others, with some being investigated for additional health issues or pursuing further diagnoses (such as ADHD). Among those who received an OI diagnosis (*n* = 7), some felt this provided a coherent explanation for their symptoms, whereas others felt at a loss to not receive the clear legitimising label of PoTS they were hoping for. The lack of perceived validation from diagnoses other than PoTS (e.g. OI, or ‘PoTSie’) some experienced, may have related to this being even less clearly defined or well-known than PoTS, which consequently may affect the treatment or psychosocial support patients receive compared to other conditions that are less defined.

Some participants, even those diagnosed with PoTS, still appeared to search for further diagnostic labels, coherence, and clarification of symptoms. The point at which the search for additional diagnostic labels becomes less helpful appeared dependent on individual factors such as pre-existing diagnoses or needing to explain sick leave at work, and some diagnostic labels (such as bladder dysfunction or reactive hypoglycaemia, which had direct management implications) were considered more useful than others (such as EDS or CFS). This is consistent with previous findings, where some labels are viewed as legitimising [13], whereas others are considered unhelpful [28,29], stigmatising [43–47], or even offensive [48]. Benefits from treatments may also be harder to discern as the treatments for different problems accumulate or, as our findings have illustrated, even clash. Ultimately, participants’ search seemed to be for an explanation for every symptom experience, whether that was from one cause or multiple, and this may become increasingly complex, the more labels that have been acquired.

Focusing on an overarching unifying explanation of multiple symptoms may reduce strain on both patients and healthcare systems, but how this could be best framed for PoTS is an area deserving of further qualitative and quantitative enquiry. Psychosocial or behavioural interventions either on their own or offered alongside medical care were not mentioned by participants, and this gap should be noted. Whilst there is a clear need for evidence-based PoTS specific treatments and clear treatment plans, these may not resolve the wider issues of managing and identifying associated co-occurring long term conditions. Moving forward, developing transdiagnostic ways of managing multiple difficult symptoms that could be offered early on, alongside specific treatments for the conditions where needed and available, may be beneficial. Exploring transdiagnostic factors for PoTS and associated co-occurring long term conditions may therefore be an important next step.

A more coherent universal, transdiagnostic treatment approach for other physical syndromes has been advocated in a move away from the purely biomedical model, which tends to treat individual symptoms, or where one is known, the underlying cause. These more cohesive tailored interventions may address unconscious inferential [49] or broadly conceptualised cognitive and behavioural aspects through manualised CBT, for example by focusing on previously identified symptoms that are overlapping across patient groups, as well as common cognitive and behavioural responses to symptoms across conditions [50,51]. Although there is some resistance and controversy regarding some of these unifying or ‘biopsychosocial’ approaches [52–54], in part due to concerns around psychologising physical illness, and perhaps particularly where there is an androcentric history of the condition being labelled as ‘hysteria’ [55], there is growing evidence that mental and physical health is non-dichotomous [56,57].

Patients, carers, researchers and care providers have long advocated a more co-ordinated, integrated approach to the management of chronic physical syndromes [58,59]. Drawing on existing treatment models may provide a starting point for developing a unique treatment model for PoTS and associated conditions. FND, which can be diagnosed and treated in the presence of co-occurring, pathophysiologically defined disease, is recommended to be managed from an integrated multidisciplinary model pertaining physiotherapy and specialised CBT [60]. This model adopts biological causes, and while psychological stressors are acknowledged as risk factors of FND, they are not always present nor recommended to be explored, to avoid re-traumatisation or being perceived as an intrusive and inappropriate search for a psychological cause [60]. This approach also proposes that considerations should be made for the care of patients, but equally at the level of healthcare systems in order to plan effectively for this group of patients, and avoid misdiagnosis, poor outcomes, iatrogenesis, frustration for patients and clinicians, and the poor use of resources [60,61]. Some of the aforementioned has been observed in PoTS patient groups [18,23] and exemplified in the current study by the unfruitful healthcare interactions [62] described by participants. Due to the problematic healthcare experiences [25] many PoTS patients have had, psychological enquiry should be avoided or handled with some delicacy. Crucially, psychosocial influences should be viewed as contributing rather than causative factors. Care for those not quite meeting PoTS HR delta criteria could be improved through early implementation of transdiagnostic or PoTS treatments for this group. Defining a separate diagnostic label for this group, or inclusion of these patients in a more widely recognised umbrella term as has been proposed in the Canadian Cardiovascular Society Position Statement [63], where the absence of sufficient tachycardia is acknowledged but not exclusive of a diagnosis, may also be warranted.

This patient group’s high and complex needs are struggling to be met by current healthcare provisions, and more timely appointments would be beneficial. Future work in this area should include interviews with HCPs to better understand the challenges and complexities of caring for this patient group.

### Trustworthiness and limitations of findings

Strengths of this study include its rigorous methodology and novelty, as this is the first study to longitudinally explore the journey to diagnosis of PoTS. Due to the Covid-19 pandemic, participants were interviewed during transitional times, where significant societal and healthcare contextual changes were taking place which may have uniquely influenced these participants’ stories, and receipt of tests and results was slower than usual. In future studies, a longer timeframe for follow-up (e.g., a whole year or more) would be merited to allow better insights into the more long term trajectory of treatments, investigations, self-management, and symptoms. Another limitation is that participants self-selected to be included and were from only one PoTS clinic, albeit a national one which attracts patients from around the country. Finally, the interviewer has lived experience of PoTS, however attempts to minimise any bias from this included using multiple coders and frequent team discussions to ensure multiple perspectives were included in the analysis.

## Conclusion

This is the first qualitative study to explore patients’ stories of obtaining a diagnosis of PoTS over time. The 6 month period showed that despite attending a specialist clinic for PoTS, for most people this did not lead to much resolution. Key positives were having a clinical expert provide validation, which contributed to acceptance of limitations and self, but for many the search for more understanding and labels continued. A unifying approach may reduce the burden on patients and healthcare systems, potentially even prior to diagnosis. This could be offered alongside, as part of, or prior to, standard medical care. However, future work is needed to identify common transdiagnostic factors to address across PoTS and its associated co-occurring long-term conditions, and to tailor existing treatment models accordingly. This should include qualitative work with HCPs to better understand the challenges of managing patients with PoTS and associated conditions. Expedited and more frequent appointments, so that patients can receive their diagnosis, trial therapies, and evaluate treatment outcomes more regularly, with adjustments made or widening out of follow-up appointments as appropriate, would be beneficial.

## Supporting information

S1 Checklist (DOCX)

S1 Appendix (DOCX)

S2 Appendix (DOCX)

S1 File (PDF)
