# Supplementary material for: One or many labels? a longitudinal qualitative study of patients’ journey to diagnosis at a specialist NHS Postural Tachycardia Syndrome (PoTS) clinic
Source: PLoS One. 2024 Jul 10;19(7):e0302723. doi: 10.1371/journal.pone.0302723 (PMC11236186; doi:10.1371/journal.pone.0302723)
Supplement: S1 Appendix — (DOCX) [file pone.0302723.s002.docx]

**Appendix 1: Interview question schedule**

| **Section 1: Misdiagnosis** | |
| --- | --- |
| Tell me about your experience of the journey to diagnosis for PoTS | *Probes*   - How long did it take to get diagnosed? - Who did you see to get diagnosed? - What other specialists have you previously seen for your PoTS symptoms (before your PoTS diagnosis)? - What other diagnoses have you have received (prior to being considered for PoTS)? - Do you feel they were accurate?     *If they describe being misdiagnosed:*   - How many times? - What with? - What effects has this had, for example on you or the treatment you have received?   *If no,*   - What do you feel you owe your timely diagnosis to?     *Follow-up only:*   - Were you diagnosed with PoTS? - Has receiving a diagnosis helped our hindered you? - In what way? - How do you feel about your diagnosis? - How do you feel in relation to your previous diagnoses since being diagnosed? |
| **Section 2: Self-management strategies** | |
| What are your experiences of managing your PoTS symptoms using non-pharmacological strategies? | - Can you give me an example of what you do to manage your symptoms on a typical day? - What are the most helpful strategies for you? - What are the least helpful strategies? - What made you decide to try it? - How effective are these strategies? (prompt: across symptoms or for specific symptoms) - What do you feel could be done to improve your self-management strategies? - What do you think should be offered - What have you been offered - What advice would give to someone else with PoTS to improve their self-management of symptoms?     *Follow-up only:*   - What is your most helpful self-management strategy now and why? - What is your least helpful self-management strategy now and why? - How did you manage your symptoms before? - How do you manage your symptoms now? - Are there any self-management strategies you haven’t tried or discontinued, and why? |
