# Supplementary material for: One or many labels? a longitudinal qualitative study of patients’ journey to diagnosis at a specialist NHS Postural Tachycardia Syndrome (PoTS) clinic
Source: PLoS One. 2024 Jul 10;19(7):e0302723. doi: 10.1371/journal.pone.0302723 (PMC11236186; doi:10.1371/journal.pone.0302723)
Supplement: S2 Appendix — (DOCX) [file pone.0302723.s003.docx]

**Appendix 2**

Coding tree of themes, central concepts and associated codes at follow-up (T2)

| **Theme** | **Central organising concepts, top level codes** | **Associated codes and sub-codes** |
| --- | --- | --- |
| Slowly moving forward and finding positive gains | Benefits from appointment or diagnosis | Being taken more seriously  Benefited from referrals  Coming to terms with problems from past  Emotionally feeling better  Empowered  Feeling positive about future  Felt alone before diagnosis  Felt isolated before  Finding meaning  Goals for the future  Good medical help  Having support  More independent  Positive impact of diagnosis   - Validation   Improving relationships  Justified  Learning to manage  Making sense  Perceptive doctor  Positive experience with HCP  Positive perception of medicine  Previously blaming themselves  Proper testing  Relief  Self-acceptance  Taken more seriously |
|  | Improvements | Improvement from antihistamines  Improvement from breathing exercise  Improvement from diet  Improvement from exercise   - HR improvement - Mental Health improvement   Improvement from medication  Improvement from mindfulness  Improvement from resting  Improvement from salt  Improvement from water  Improvement in BP  Physically better |
|  | Retrospection | Looking back on relations with sceptical family  Reflecting on previous events and symptoms in their life  Stopped blaming themselves  Trying to fix relationship with mother |
|  | Factors influencing the journey | Working in HC  Private HC |
|  | Behaviours, Beliefs, Attitudes & Perceptions influencing the journey and self-management | Boom and crash  Fluctuating eating patterns  Focusing on everything  Viewing things in a positive light  Comparing herself  Doesn't think diagnosis will be useful  Not convinced it is PoTS  Not expecting diagnosis |
|  | What participants seek or would find beneficial | Fact sheet for what you could do for PoTS/EDS/comorbidities  Financial support  Information about safety of self-management  Information sheet  More funding needed  Needs diagnosis for life insurance  Physical symptoms should be priority  Prescribed exercise would be useful  Useful if receives diagnosis   - Useful for work - Useful to receive medication - Useful if receives more guidance   Validation  Wanting reassurance  Wanting to get a routine  Wanting to understand symptoms  Wants help to reduce physical symptoms  Wants to get on with life  Wants to receive help  Would benefit from increased services |
| Needing more pieces of the puzzle to see the bigger picture | Coherence | Confusion over diagnostic labels and test results  Confusion over symptoms  Covid as a potential trigger  Doing own research  Other labels hindering access  Overlap between conditions  Potential underlying cause  Prefers diagnostic labels  Previous stroke  Stress-related  Trauma in childhood  Understanding the connection between conditions |
|  | Lack of communication | Conflicting advice given to patients  Confusion over investigations  Guidance would be useful  Lack of awareness of PoTS  Lack of diagnosis means difficulty telling work  Lack of expertise  Lack of trust in HCPs  Meeting criteria   - Uncertainty over diagnosis |
|  | Negative HC experiences | Avoiding the GP  Delays   - Frustration over not seeing the right people - Waiting for medication - Waiting times for appointments   Dismissive attitudes  Misattributing symptoms  Not a lot of advice from HCP  Not feeling heard  Reduced access to services |
|  | Questioning HC, self-management strategies and investigations | Negative perception of advice  Not understanding why water helps  Safety of Self-Management strategies  Sceptical  Querying tilt table testing |
|  | Not meeting criteria | Feeling uncertain |
| The value and impact of investigations  (and the ‘*cumulative burden*’, continued from T1 analysis) | Emotional impact | Feeling like they are drowning  Feels defeated  Feels worried when going out  Finding it difficult to adapt  Guilt over feeling so tired |
|  | The value of proper testing | Importance of having symptoms investigated and taken seriously |
|  | Finding new problems | Discovering health problems they were not previously aware of |
|  | Family influences | Family background in HC  Child’s health problems  Family medical history  Family support  Positive impact of seeing family |
|  | Impact on daily life | Burden of appointments  Balance between work and symptoms  Financial impact of condition  Impact of being housebound  Impact on daily functioning  Not living independently  Not telling job  Reducing hours at work  Slowly returning to work  Socialising  Still working  Studying  Support from work  Using holiday days off for appointments |
|  | Specialists seen | Endocrinologist  ENT suggested PoTS  Headache specialist |
|  | Medication | Angina medicine  Antidepressants  Antihistamines  Antivirals  Avoid medication  Midodrine  Placebo effect  Prescribing electrolytes  Prochlorperazine  Recommended medication  Negative perception of side effects  No improvement from dizziness medication |
|  | Self-management strategies | Alternative therapies  Avoid online advice  CFS treatments  Compression tights  Conflict between symptoms and self-management  Diet   - Diet causing problems with cholesterol   Does not use online support groups  Exercise  Exercise worsening ME  Finding solutions themselves  FitBit to monitor HR  Fluids   - Water only fasts   Footstool when working  Levine Protocol  Lifestyle changes   - Making house more accessible - Sleep   Lying down  Lying down does not help  Meditation  Mindfulness  Pacing yourself  Not pushing themselves  Pushing herself negative  Pushing herself positive  Reading  Online advice  Social media  Peer support  Personal Trainer  Planning and being aware  Respiratory physiotherapy  Resting  Routine  Running   - Not sit down after running   Salt  Swimming  Trying to find a balance between PH and MH  Unsure if Self-Management is working  Walking stick  Wrist supports |
|  | Symptoms | Bed Bound  Bladder problems  Brain fog  Decline in energy levels  Dizziness  Fatigue  Fluctuating symptoms  High cholesterol  High cortisol levels  High heart rate  High prolactin levels  Joint pain  Lung problems  Nausea  Onset of symptoms  Orthostatic symptoms  Possible infection  Postural changes  Potential food allergies  Problems in summer  Problems standing  Problems walking  Problems with blood pressure  Problems with leg and blood flow  Propensity to allergies  Reduced capacity  Fatigue  Seizures  Side effects of antidepressants  Sugary drops  Symptoms do not interfere  Symptoms presenting difficulties to do work  Unpredictable symptom flares  Using arms is difficult  Weakness  Worsening of symptoms |
|  | Co-occurring conditions | ADHD  Adrenergic PoTS  Anxiety Symptoms  Autoimmune problems  Chronic sympathetic activation as a possible cause of PoTS  COVID  EDS / Hypermobility  Fibromyalgia  IBS issues  MCAS  ME (CFS) diagnosis   - ME impacting daily functioning   Polychondritis  Reactive hypoglycaemia |
|  | Tests & procedures conducted | Blood tests  Brain MRI  Cardiopulmonary exercise testing  Chest Clinic  Heart MRI  Lung function tests  Operations  Does not like tests  ECG  Tilt test  Urine test for salt |
