# Supplementary material for: One or many labels? a longitudinal qualitative study of patients’ journey to diagnosis at a specialist NHS Postural Tachycardia Syndrome (PoTS) clinic
Source: PLoS One. 2024 Jul 10;19(7):e0302723. doi: 10.1371/journal.pone.0302723 (PMC11236186; doi:10.1371/journal.pone.0302723)
Supplement: S1 File — (PDF) [file pone.0302723.s004.pdf]

Aims:

To investigate patients' journey to diagnosis at a specialist NHS PoTS clinic

Methods:

Qualitative longitudinal reflexive thematic analysis

Results:

Three superordinate themes detailed below

Slowly moving forward and finding positive gains

Patients found validation and legitimisation of their symptoms in a helpful specialist appointment and diagnosis. This promoted acceptance of limitations posed by the symptoms.

Needing more pieces of the puzzle to see the bigger picture

An explanation for every symptom experience was sought, and in the context of multiple co-occurring conditions, this may become increasingly complex, the more diagnoses that have been acquired.

The value and impact of investigations

Investigations, appointments, and new-found problems had a substantial impact over time on the cumulative burden of symptoms, treatments, co-occurring conditions, and psychosocial consequences.
